# The endocannabinoid 2-arachidonoylglycerol mediates seasonal life-history transitions in a wild reptile

**DOI:** 10.1242/jeb.251845

**Published:** 2026-07-06

**Authors:** Lauren J. Merlino, Pauline Florence, Lin Lin, Faizy Ahmed, Daniele Piomelli, Deborah I. Lutterschmidt

**Affiliations:** ^1^ Department of Ecology and Evolutionary Biology, University of California, Irvine, Irvine, CA 92697, USA; ^2^ Department of Anatomy and Neurobiology, University of California Irvine, Irvine, CA 92697, USA; ^3^ Department of Biological Chemistry, University of California Irvine, Irvine, CA 92697, USA; ^4^ Department of Pharmaceutical Sciences, University of California Irvine, Irvine, CA 92697, USA

**Keywords:** Courtship behavior, Migration, Locomotion, Foraging, Snake, Glucocorticoid

## Abstract

The mechanisms regulating seasonal transitions between reproduction and feeding behavior are not well understood. Endocannabinoids modulate neuronal signaling in reproductive, metabolic and locomotive control centers and are widely distributed throughout the vertebrate nervous system, making them a strong candidate for facilitating seasonal transitions. Using liquid chromatography tandem mass spectrometry, we asked whether brain endocannabinoid concentrations in wild red-sided garter snakes (*Thamnophis sirtalis parietalis*) vary with behavioral phenotype, migratory status or sex. We found that levels of 2-arachidonoylglycerol (2-AG) in the reptilian homolog of the mammalian hippocampus decreased significantly as male and female snakes began their migration to feeding grounds. To further evaluate whether endocannabinoids mediate seasonal behavior, we manipulated endocannabinoid signaling for 14 days in males with daily intraperitoneal injections of vehicle control or two different doses of cannabinoid 1 receptor antagonist (AM251), cannabinoid receptor agonist (CP-55940) or an inhibitor of the 2-AG degradative enzyme monoglyceride lipase (JZL184). We found that blocking cannabinoid 1 receptor with AM251 (15 μg per snake; approximately 0.5 mg kg^−1^) significantly reduced male courtship behavior, but not locomotor activity, after 7 days of treatment. Despite 8 months of hibernation-associated aphagia, increasing endogenous levels of 2-AG with JZL184 (48 µg per snake; approximately 1.6 mg kg^−1^) delayed the onset of food-motivated behavior and extended reproductive behavior in males. Our results support a role for 2-AG in the seasonal maintenance and termination of reproductive behavior. Because the endocannabinoid system is evolutionarily conserved across vertebrates, these data are broadly applicable to understanding the neuroendocrine mechanisms that mediate trade-offs between reproduction and self-maintenance.

## Teaser

**Summary:** 2-Arachidonoylglycerol (2-AG) decreases in the reptilian hippocampus during the seasonal transition from mating to feeding behavior; pharmacological manipulations demonstrate that signaling via cannabinoid receptor 1 mediates this behavioral transition.

## INTRODUCTION

All animals must navigate trade-offs between self-maintenance and reproduction to maximize both survival and fitness. A common feature of these trade-offs is engaging in specific behaviors when necessary resources to support a given behavior are most available. Because resources such as mates, food and suitable habitat vary both seasonally and geographically, many animals undergo dramatic shifts in behavior as they transition between reproductive and self-maintenance opportunities (e.g. migrating between breeding sites and foraging areas). The mechanisms that mediate these life-history transitions remain poorly understood, in part because of challenges associated with interpreting behavior of laboratory animals in an ecological context and in part because of historically limited toolsets for studying wild animals. However, the near-concurrent timing of changes in appetitive behavior toward different resources (i.e. motivation for one resource wanes while motivation for another resource waxes) suggests mechanisms involving neuroendocrine modulation and/or neuroplasticity are involved.

The endocannabinoid system is a strong candidate for mediating seasonal life-history transitions, as it is involved in the neuromodulation of several relevant physiological processes, including appetite (Kirkham, 2005), feeding (Di Marzo and Matias, 2005), nutrient metabolism (Watkins and Kim, 2014), reproduction (Meccariello et al., 2014), motor control (El Manira et al., 2008), spatial memory (Figueiredo and Cheer, 2023), synaptic plasticity (Chevaleyre et al., 2006) and decision making (Hernandez and Cheer, 2015). For example, both endocannabinoids and plant-derived cannabinoids, such as Δ9-tetrahydrocannabinol (THC), are well known for their ability to stimulate hunger and increase food intake. The orexigenic effects of endocannabinoids have been described in humans (reviewed by Aguilera Vasquez and Nielsen, 2022), rodents (Kirkham et al., 2002), ungulates (Van Ackern et al., 2021), birds (Alizadeh et al., 2015; Keyshams et al., 2016) and fish (Cottone et al., 2009; Diaz-Rua et al., 2021; Valenti et al., 2005), although the direction of those effects is dose dependent in some species. Furthermore, evidence of an endocannabinoid system has been identified in all vertebrate classes [mammals (Herkenham et al., 1990), birds (Soderstrom and Johnson, 2001), reptiles (Astarita et al., 2006), amphibians (Soderstrom et al., 2000), fish (Yamaguchi et al., 1996)] and many invertebrates, including echinoderms (Chang et al., 1993), mollusks (Sepe et al., 1998), annelids (Stefano et al., 1997), cnidarians (De Petrocellis et al., 1999) and insects (Jeffries et al., 2014; Zhong et al., 2024; but also see McPartland et al., 2001), suggesting that the presence and possibly function of the endocannabinoid system is evolutionary conserved.

The endocannabinoid system consists of endogenous lipid-based ligands and their receptors. The synthesis of endocannabinoids is modified by catabolic/anabolic enzymes that, together with their receptors, comprise complex signaling pathways known to interact with other neuroendocrine factors, particularly within hypothalamic axes (reviewed by Pagotto et al., 2006; Hillard, 2015). The best studied endocannabinoids are 2-arachidonoylglycerol (2-AG) and anandamide. Unlike classic neurotransmitters, these lipid-derived messengers are not stored in vesicles but instead are produced on demand. Specifically, 2-AG is produced by diacylglycerol lipase isoform α (DGLα) and hydrolyzed by monoacylglyceride lipase (MGL), while anandamide is produced by *N*-acyl phosphatidylethanolamine phospholipase D (NAPE-PLD) and hydrolyzed by fatty acid amide hydrolase (FAAH) (reviewed by Piomelli and Tagne, 2022). After being released from cells, endocannabinoids act through autocrine or paracrine mechanisms, sometimes acting as retrograde messengers by diffusing backwards across the synapse from the postsynaptic terminal to presynaptic receptors.

Endocannabinoids primarily bind to cannabinoid 1 (CB1) and cannabinoid 2 (CB2) receptors, both part of the G protein-coupled receptor family. While CB1 receptors are abundantly distributed throughout the vertebrate central nervous system and body, CB2 receptors tend to be found in lower densities and are mostly, although not exclusively, found on immune cells (reviewed by Howlett and Abood, 2017). When endocannabinoids bind to CB1 receptors, they initiate a series of second messenger signaling pathways that can close voltage-gated Ca^2+^ channels, open K^+^ channels and/or inhibit adenylyl cyclase, ultimately reducing the release of neurotransmitters, often excitatory glutamate or inhibitory GABA (reviewed by Piomelli, 2003). The ability to rapidly mediate synaptic transmission, combined with the ubiquity of endocannabinoid receptors throughout the body, allows endocannabinoids to regulate a wide range of neuroendocrine communication. Although endocannabinoids are known to mediate neuroendocrine processes related to sex behavior (reviewed by Rodríguez-Manzo and Canseco-Alba, 2023) and food intake (reviewed by Aguilera Vasquez and Nielsen, 2022) in laboratory animals, it remains largely unknown how they function in wild animals. Surprisingly, only one study to date has investigated endocannabinoid signaling in relation to sex behavior in a wild animal (Coddington et al., 2007), and no study has explored endocannabinoid signaling in relation to feeding behavior in any wild animal.

Garter snakes are a powerful comparative study organism where the relationship between endocannabinoid signaling and seasonal sex and feeding behavior can be isolated, readily interrogated, and interpreted within the context of the animal's ecology. Northern populations of red-sided garter snakes hibernate in underground dens for 8 months, where a single hibernaculum can have upwards of 30,000 snakes (Shine et al., 2006). In late April and early May, males emerge from their underground den and form lek-like aggregations (Aleksiuk and Gregory, 1974). Males begin to emerge 1–2 weeks prior to females and remain at the den for several weeks as they continue their search for mates (Gregory, 1974; Shine et al., 2001). Following emergence, a single female can be courted by as many as 100 males within one mating ball prior to migrating away from the den within 24 h of emergence (D.I.L., R. C. Wilson and M. P. LeMaster, unpublished data; Gregory, 1977; Joy and Crews, 1985; Shine et al., 2006). Male and female snakes migrate from the den in all directions, traveling up to 18 km to find abundant food (Gregory and Stewart, 1975). Importantly, courting males collected from the den during the spring mating season do not eat, even when offered food (O'Donnell et al., 2004), suggesting the switch to migratory and feeding behavior is endogenously regulated.

In this study, we designed three experiments to investigate whether endocannabinoids play a role in regulating the transition from reproduction to feeding behavior. More specifically, we asked whether levels of brain endocannabinoids are associated with (1) changes in reproductive behavior and/or (2) the initiation of spring migration to feeding grounds. Finally, we experimentally manipulated endocannabinoids in male snakes to determine (3) whether endocannabinoids directly influence courtship and/or food-motivated behavior.

## MATERIALS AND METHODS

### Animal care and use

All experiments were conducted in the field during the spring mating season of red-sided garter snakes, *Thamnophis sirtalis parietalis* (Say 1823), located in the Interlake region of Manitoba, Canada. Reproductively mature, adult snakes were collected from their den or a road ∼1 km away from the den. Snakes were housed in groups, separated by sex, in semi-natural cylindrical outdoor arenas (48 cm diameter, 1 m height) with a hide box and access to water. These housing conditions do not affect baseline corticosterone or androgen levels, nor do they induce a stress response (Cease et al., 2007; Lutterschmidt and Maine, 2014; Moore and Mason, 2001).

Protocols for experiments 1 and 3 were approved by the University of California, Irvine Institutional Animal Care and Use Committee (protocols AUP-22-035 and AUP-25-037) and performed under the authority of Wildlife Scientific Permit WB25900 issued by the Manitoba Department of Natural Resources and Indigenous Futures. Tissues analyzed for endocannabinoids in experiment 2 were collected as part of a separate study examining possible variation in liver glycogen and adipocyte area with sex and migratory status (Wilson and Lutterschmidt, 2020). These protocols were approved by the Portland State University Institutional Animal Care and Use Committee (Protocol #40) and performed under the authority of Wildlife Scientific Permit WB18801 issued by the Manitoba Department of Natural Resources and Indigenous Futures.

### Experimental design

#### Experiment 1

In experiment 1, we asked whether there is variation in the endocannabinoids 2-AG and anandamide in the brains of male red-sided garter snakes exhibiting different behavioral phenotypes. To answer this question, we kept migratory status constant while focusing on possible changes associated with shifts in mating and feeding behavior. We collected non-migratory male snakes (*n*=17) from the den and housed them in semi-natural arenas until behavioral assays were performed. To identify the behavioral phenotype, we first measured reproductive behavior using a well-established ethogram (from Lutterschmidt et al., 2004; see ‘Reproductive behavior’, below, for additional details). A male displaying chin-rubbing behavior (a minimum courtship score of 2) during the trial was characterized as a courting male, as males only exhibit this behavior in a reproductive context. We then assessed feeding behavior with a two-choice Y-maze assay to determine whether males prefer to follow a worm trail (feeding opportunity) or a female pheromone trail (reproductive opportunity) when given the choice between the two (see ‘Feeding behavior’, below, for additional details). Together, these two behavioral assays were used to determine a male snake's behavioral phenotype (Table 1). After identification of their behavioral phenotype, snakes were euthanized with 300–500 μl of 1% sodium Brevital injected near the heart and whole brains were immediately microdissected and frozen on dry ice for later analysis.

**Table 1.** Behavioral phenotypes associated with the transition from spring mating to summer feeding

Behavioral phenotype	Courtship trial results	Two-choice Y-maze results
Sex motivated	Courted female	Chose female trail over worm trail
Transitional	Courted female	Chose worm trail over female trail
Food motivated	Did not court female	Chose worm trail over female trail

A male's behavioral phenotype was assessed using the combined results of a courtship trial and a two-choice Y-maze assay.

#### Experiment 2

In experiment 2, we asked whether there is variation in the endocannabinoids 2-AG and anandamide in the brains of male and female snakes with different migratory status during the spring mating season. Similar to Cease et al. (2007) and as described in Wilson and Lutterschmidt (2020), reproductive, non-migratory snakes were collected from the den site where mating occurs (*n*=16 males, *n*=15 females). Migratory snakes (*n*=16 males, *n*=12 females) were collected at the beginning of their seasonal migration to summer feeding grounds by intercepting snakes as they crossed a rural road along their migration route, approximately 1 km from the den. In these experiments, migratory status is considered a proxy for changes in motivation and the transition to feeding behavior. Before migration, male snakes at the den are interested in mating and refuse food when offered (Crews et al., 1987; O'Donnell et al., 2004). At the beginning of their migration, some males begin to show interest in food (based on their trailing behavior and anecdotal evidence of gut contents; e.g. Cease et al., 2007), but the majority will still refuse to eat. Snakes were euthanized immediately after capture and blood sample collection with 300–500 μl of 1% sodium Brevital injected near the heart. Brains were immediately microdissected on ice into two gross brain regions, the diencephalon and telencephalon, for each brain hemisphere and frozen on dry ice for later analysis. The mean time elapsed between capturing a snake and freezing tissues on dry ice was less than 10 min. Within the diencephalon, the primary neuroendocrine structure is the hypothalamus. The telencephalon region includes the dorsal, medial and lateral cortex and the nucleus sphericus. The medial and dorsal cortex of reptiles is a structural and functional homolog of the avian and mammalian hippocampus, while the nucleus sphericus is homologous to the medial amygdala (Butler and Hodos, 2005). For ease and clarity, we will refer to the diencephalon and telencephalon as the hypothalamus and hippocampus, respectively.

#### Experiment 3

In experiment 3, we asked whether manipulating endocannabinoid signaling affects courtship and feeding behaviors associated with the life-history transition from spring mating to summer feeding. We collected courting male snakes (*n*=126) from mating balls at the den; all snakes exhibited tail wrestling behavior (i.e. had a courtship ethogram score of 4; from Lutterschmidt et al., 2004). We also confirmed their trailing preference for female pheromone cues rather than worm trails on the Y-maze, thereby ensuring that all snakes started with the same behavioral phenotype. Snakes were randomly divided among seven treatments that alter endocannabinoid signaling (*n*=18 for each treatment group, *n*=126 total): vehicle control, two doses of AM251, a CB1 receptor antagonist, two doses of JZL184, an inhibitor of the 2-AG degradation enzyme MGL, or two doses of CP-55940, a CB1 receptor agonist (see ‘Drug preparation and administration’ for exact dosages used in this experiment). Snakes received intraperitoneal treatment injections daily for 14 days and their behavior was systematically evaluated throughout the experiment. Injections were administered between 08:00 h and 10:00 h each day. On days 3, 7 and 12, we measured reproductive behavior using courtship trials. During the courtship trials on day 3, we observed variation in locomotor ability across treatment groups. Thus, for the courtship trials on days 7 and 12, we incorporated a three-point locomotion score to evaluate whether manipulating endocannabinoid signaling altered locomotor function in snakes (see ‘Locomotor behavior’, below, for more details). On days 5, 10 and 15, snakes' motivation for feeding opportunities was evaluated using a two-choice Y-maze. Finally, on days 0, 7 and 14, snakes were weighed to determine changes in body mass over time. At the end of the experiment, all snakes were released at their site of capture.

### Lipid extraction

We extracted 2-AG and anandamide as previously described (Ahmed et al., 2024; Torrens et al., 2020; Vozella et al., 2019). In randomized order, frozen brain samples were weighed and transferred to Precellys^®^ CK-14 soft tissue tubes (Bertin Technologies, Paris, France) with 500 μl of ice-cold acetonitrile containing 1% formic acid and then spiked with 50 μl internal standard mix (500 pmol [^2^H]-2-AG, 100 pmol [^2^H]-anandamide). Brains were homogenized with a Precellys Evolution Homogenizer (Bertin Technologies) twice for 15 s at 4°C and centrifuged for 15 min at 1301 ***g*** at 4°C. Captiva-Enhanced Matrix Removal (EMR)-Lipid cartridges (Agilent Technologies, Wilmington, DE, USA) were prewashed with 200 μl water:acetonitrile (1:4 v/v) and run under vacuum (3–5 mmHg). Sample supernatants were loaded into the EMR cartridges and eluted under vacuum. The remaining tissue pellets were rinsed with 200 μl water:acetonitrile (1:4, v/v) and mixed for 30 s before being centrifuged for 15 min at 1301 ***g*** at 4°C. The supernatants were then loaded into the cartridges, eluted, and pooled with the first eluate. All cartridges were washed a final time with 200 μl water:acetonitrile (1:4, v/v) under vacuum pressure that gradually increased to 10 mmHg for maximal analyte recovery. The pooled eluates were dried under nitrogen gas, reconstituted in 100 μl of methanol, vortexed and transferred to deactivated 0.2 ml glass inserts inside 2 ml amber glass vials. Samples were frozen at −80°C until lipid quantification using liquid chromatography tandem mass spectrometry (LC-MS/MS) analysis.

### Lipid quantification

Stock solutions containing 2-AG and anandamide were prepared and serially diluted in methanol to generate a calibration curve, where their [^2^H]-containing internal standards were added to each point (Cayman Chemical, Ann Arbor, MI, USA). Agilent's MassHunter software normalizes analyte abundance in ratio to internal standard abundance to produce a calibration curve. Internal standard ion peaks must be optimized to the ion peak of the sample (Wang et al., 2017). Because endocannabinoids have never been quantified in brain tissue of snakes, the estimated ion peak of our samples was unknown. Thus, we ran experiment 1 using an external calibration curve without an internal standard. Those results allowed us to estimate internal standard concentrations used for experiment 2. Importantly, endocannabinoid concentrations from experiment 1 and 2 are comparable. Calibration curves for both experiments can be viewed in Fig. S1.

Sample analyses were performed similar to those described in Fotio et al. (2024). 2-AG and anandamide were analyzed by LC-MS/MS using a 1260 series LC system consisting of a binary pump, degasser, autosampler with a chiller and a thermostated column compartment coupled to an Agilent 6460C triple quadrupole mass spectrometric detector with a Jet Weaver™ electrospray ionization interface (Agilent Technologies). Endocannabinoids were chromatographically separated using an Agilent ZORBAX^®^ Eclipse PAH C18 column (1.8 μm, 2.1×50.0 mm; Agilent Technologies). The mobile phase consisted of 0.1% formic acid in water as solvent A and 0.1% formic acid in methanol as solvent B. The flow rate was 0.3 ml min^−1^. A linear gradient was used: 75% solvent B at 0 min to 80% solvent B at 5.00 min, 95% solvent B at 5.01 min continuing to 6.00 min, and 75% solvent B at 6.01 min continuing to 8.00 min, the total run time. The column temperature was maintained at 45°C and the autosampler at 10°C. The injection volume was 2.0 μl. To prevent carry over, the needle was washed in the autosampler port for 10 s before each injection using a wash solution consisting of 10% acetone in a solution consisting of water:methanol:isopropanol:acetonitrile (1:1:1:1, v/v).

The mass spectrometric detector was operated in the positive electrospray ionization (ESI) mode. Multiple reaction monitoring (MRM) mode was used with the following ion transitions and their associated fragmentor (frag) voltage and collision energy (CE) voltage: 2-AG-^2^H_5_ 384.3→287.2 (frag 125 V, CE 10 V), 2-AG quantifier 379.3→287.2 (frag 123 V, CE 10 V), 2-AG qualifier 379.3→269.2 (frag 123 V, CE 13 V), anandamide-^2^H_4_ 352.3→66.2 (frag 140 V, CE 50 V), anandamide quantifier 348.3→62.2 (frag 128 V, CE 13 V), anandamide qualifier 348.3→91.1 (frag 128 V, CE 45 V). The drying and sheath gas temperatures were 300°C each with flow rates of 5.0 ml min^−1^ and 12 ml min^−1^, respectively. Capillary and nozzle voltages were set at 3000 V and 1900 V, respectively.

**Fig. 1.**
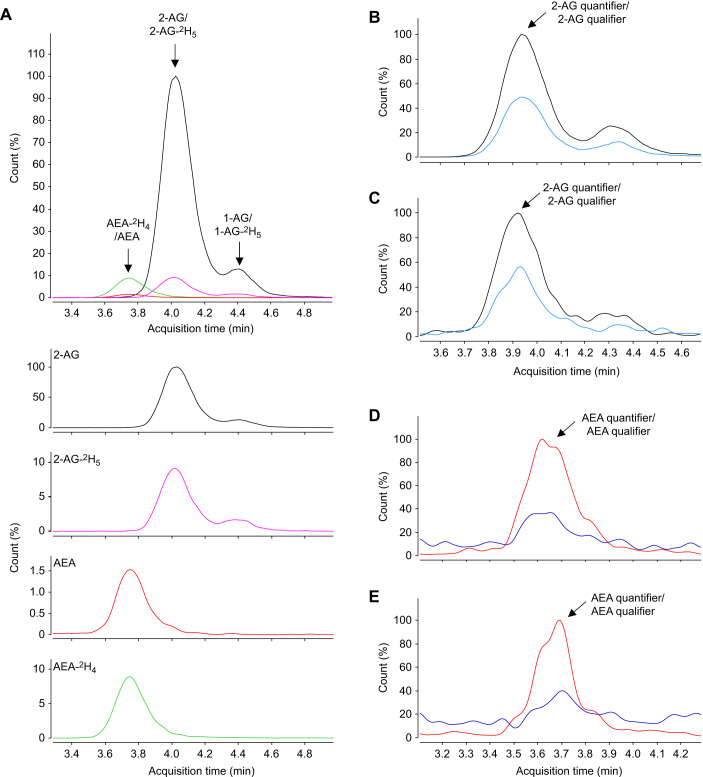
**Identification of the endocannabinoids 2-arachidonoylglycerol and anandamide in the brain of wild-caught red-sided garter snakes.** (A) Representative liquid chromatography separation of 2-arachidonoylglycerol (2-AG), 1-arachidonoylglycerol (1-AG), anandamide (AEA) and their [^2^H]-containing internal standards from the garter snake brain. (B,C) Separation of 2-AG quantifier and qualifier ions in (B) garter snake brain and (C) synthetic 2-AG standard at the lowest point on its calibration curve. (D,E) Separation of AEA quantifier and qualifier ions in (D) garter snake brain and (E) synthetic AEA standard at the lowest point on its calibration curve.

Sample analyses were performed in two replicates and averaged. Analyte concentrations were normalized to brain tissue mass (mg) to calculate a final concentration of pmol mg^−1^ or fmol mg^−1^. Ion abundances of 2-AG and its analog 1-arachidonoylglycerol (1-AG) were added together per standard practice to determine total 2-AG, as 1-AG originates from biologically active 2-AG via acyl migration (Hillard, 2018). Agilent MassHunter software (Agilent Technologies) was used for instrument control, data acquisition and analysis. A representative chromatogram showing the separation of 2-AG and anandamide, their [^2^H]-containing internal standards and their qualifier ions is shown in Fig. 1.

### Enzyme assay to validate the effects of JZL184 in snake tissue

Prior to conducting experiment 3, we confirmed that JZL184 increases 2-AG concentration in isolated garter snake tissue, as it is known to do in mice (Pan et al., 2009). We homogenized brain tissues from 5 male snakes (collected as part of an independent, unrelated experiment) individually in 1 ml of ice-cold reptile Ringer's solution; 800 μl from each brain homogenate was then combined into one pooled sample and further diluted with 4 ml of ice-cold reptile Ringer's solution. We used this pooled sample to validate the effects of JZL184 (Cayman Chemical) compared with a vehicle control at two different temperatures: a mammal-appropriate temperature (37°C) and a reptile-appropriate temperature (24°C, which approximates the preferred body temperature of red-sided garter snakes during the spring mating season and is below their critical thermal maximum; Hubert, 2022).

For each of the 4 treatment conditions (2 treatments×2 temperatures), we used the pooled sample to generate 3 replicates containing 400 μl each, which were kept on ice. We then added 80 μl of vehicle (final concentration 10% DMSO in reptile Ringer's solution) or JZL184 (final concentration 5 μmol l^−1^ JZL184 in vehicle) to each replicate and vortexed it twice for 15 s each. Each replicate was then immediately divided into four 120 μl aliquots to assess changes in 2-AG levels over time. Aliquots were placed into syringe-top vials to prevent evaporation. Incubation started promptly when samples were placed in either 24 or 37°C water baths. After 30, 60, 120 or 240 min, aliquots were removed from their water baths and immediately quenched with 500 μl of ice-cold acetonitrile containing 1% formic acid, the first step in lipid extraction for LC-MS/MS analysis. Lipid extraction continued as described above, except samples dried under nitrogen gas were also heated to 35°C to evaporate the DMSO. Samples were frozen at −80°C until lipid quantification of 2-AG was performed, as described above.

### Drug preparation and administration

All drugs for experiment 3 were prepared fresh each day immediately prior to injection. AM251 (Cayman Chemical), JZL184 (Cayman Chemical) and CP-55940 (Sigma-Aldrich, St Louis, MO, USA) were dissolved in a vehicle solution of DMSO:PEG-400:Tween-80:reptile Ringer's solution (20:10:10:60, v/v/v/v) with gentle heat (35°C) and sonication. Drug and vehicle treatments were administered by intraperitoneal injection of 100 μl containing 15 or 150 μg AM251, 48 or 480 μg JZL184, or 3 or 30 μg CP-55940. For an average male snake weighing 30 g, this produced treatment doses that were approximately 0.5 or 5 mg kg^−1^ AM251, 1.6 or 16 mg kg^−1^ JZL184, or 0.1 or 1 mg kg^−1^ CP-55940. For ease and comparability with other studies, we will refer to these doses as their approximate value in mg kg^−1^. The high doses are comparable to those used in mammals (Chambers et al., 2006; Hamamoto et al., 2007; Kinsey et al., 2013), and the low doses are 10-fold lower to account for slower metabolism in ectotherms.

### Reproductive behavior

Behavioral assays were performed in nylon cloth arenas (1×1×1 m) to evaluate male reproductive behavior, with 14–25 male snakes randomly assigned to each arena. Male red-sided garter snakes display facilitated courtship behavior, where they are attracted to females by both the pheromone on the female's dorsal surface and the formation of a mating ball (Joy and Crews, 1985). Snakes from each treatment group were equally distributed across arenas. Males in each arena were given a unique color marking with permanent marker on their dorsal stripe to allow for easy identification during behavioral assays. The observer was blind to the treatment group of each snake.

After a 20 min acclimation period, we introduced two sexually attractive stimulus females into each arena [i.e. females that express the sex attractiveness pheromone and elicit courtship from males, as outlined by Garstka and Crews (1981) and Mason et al. (1989)]. We quantified male courtship behavior at 10, 20 and 60 min after introduction of stimulus females using a well-established ethogram {scores range from 0 to 5, where a 5 is copulation [from Lutterschmidt et al. (2004); modified from Moore et al. (2000) and Crews et al. (1984)]}. Because female red-sided garter snakes become unreceptive and unattractive to males after copulation (Garstka et al., 1982), the cloaca of each female was covered with medical adhesive tape prior to the courtship trial. Neither male nor female reproductive behavior is altered by the adhesive tape (Lemaster and Mason, 2002; Lutterschmidt and Mason, 2008) and it was removed immediately following each trial. As a result of the tape, a courtship score of 4 (corresponding to tail wrestling and caudocephalic waves) was the highest score a male could attain. For each male, courtship scores within each trial were used to calculate his mean courtship score on days 3, 7 and 12 of the experiment. All courtship trials were conducted during the snakes' normal activity period between 10:30 h and 15:30 h.

### Locomotor behavior

Locomotor behavior was evaluated simultaneously with courtship behavior on days 7 and 12 of experiment 3. Each time a courtship score was assigned during the courtship trial (i.e. at 10, 20 and 60 min), locomotor activity was also assessed using a three-point locomotion ethogram. In this ethogram, a score of 0 indicated no locomotor activity; 1, general locomotor activity but not including courtship behavior towards a female; 2, locomotor activity associated with actively courting a female. This scoring system allowed us to assess the effects of endocannabinoid manipulations on appetitive reproductive behavior separately from those on general locomotor activity.

### Feeding behavior

To identify when male snakes transitioned from appetitive sex behavior to appetitive feeding behavior, we used a two-choice Y-maze trial as described in Lutterschmidt and Maine (2014) and O'Donnell et al. (2004). Butcher paper was used to cover the surface of the Y-maze and straws were used to cover the vertical pegs, all of which were replaced daily to remove residual chemical cues. Before the start of each experiment, a bias test was performed to ensure there was no preference for either Y-maze arm in the absence of stimuli (both maze arms blank). We then baited the Y-maze with a worm trail (a feeding cue) and a female pheromone trail (a reproductive cue). Earthworms were collected from the Chatfield Research Station in Manitoba, Canada, as red-sided garter snakes regularly eat them (Cease et al., 2007; Gregory, 1974). To create a worm trail, an earthworm was dragged along the butcher paper from the base of the Y-maze up one arm. The female trail was made by rubbing the dorsal surface of a sexually attractive stimulus female on the butcher paper from the base of the Y-maze up the other arm. Worm and female trails crossed at the Y-junction, ensuring that each male always came in contact with both trails before making a choice. Trail arm assignment was randomized.

Each trial began by placing a randomly selected male into an opaque box with an opening onto the base of the Y-maze, allowing the snake to enter the maze of their own accord. If males did not leave the box after 5 min, the box lid was opened slightly to encourage them to leave. If males did not leave the box after 10 min, the box lid was slowly removed. Snakes exited the trial when their head passed the last vertical peg at the end of either arm. We recorded the trail choice of each male and their latency to choose in experiment 3. We noted displays of trail contact behavior, characterized by overt investigation of the trail with short, rapid tongue flicks immediately followed by movement along the female or worm trail (Brown and MacLean, 1983; LeMaster and Mason, 2001). Notably, trail contact responses to female pheromone trails often include chin rubbing the paper and caudocephalic waves, both characteristic of male courtship behavior. Trail contact responses to worm trails include tongue flicking the trail, searching behavior and sometimes biting at the worm trail.

Because of the 15 day time course and large sample sizes in experiment 3, we limited males to 20 min on the Y-maze. If a male snake did not leave the box or move up the Y-maze within 20 min, their trial ended and they were assigned a latency to choose of >20 min. If after 20 min a male snake had not made a choice but was still moving, they were allowed to continue moving until they either made a choice or stopped moving, at which point their trial ended and they were assigned a latency to choose of >20 min. All Y-maze trials were conducted during the snakes' normal activity period between 09:00 h and 18:00 h.

### Statistical analysis

Unless otherwise noted, all data were normally distributed and exhibited equal variance among groups or were transformed to meet the assumptions for parametric tests. Significant main effects detected by analysis of variance (ANOVA) were further examined using a Student–Newman–Keuls multiple comparisons test, which utilizes step-down logic (Zar, 2010). We chose this multiple comparisons test because we had *a priori* knowledge that courtship behavior and body mass would exhibit a step-wise decrease over time. When a significant interaction was observed, we report results of multiple comparisons tests within each factor, as a significant interaction indicates the effects of one factor depend on the level of the other factor. For repeated measures analyses, assumptions of sphericity were either met or violated, in which case a Greenhouse–Geisser correction was used. Statistical comparisons were considered significant at *P*≤0.05. Effect sizes are reported for ANOVA and chi-squared results as eta-squared (η^2^) or Cramer's *V*, respectively. All statistical analyses and figures were produced using SigmaPlot 14.5 (Grafiti LLC, Palo Alto, CA, USA) and SPSS 29 (SPSS Inc., Chicago, IL, USA).

In experiment 1, a one-way ANOVA was used to determine whether endocannabinoid concentrations in whole-brain samples varied significantly among behavioral phenotypes. In experiment 2, we used a two-way ANOVA to determine significant main effects of sex and migratory status on endocannabinoid concentrations within each brain region. For both experiments, final endocannabinoid concentrations were normalized to the mass of the extracted brain tissue to account for potential allometric effects associated with sexually dimorphic body size and indeterminate growth in garter snakes.

In experiment 3, we first confirmed that JZL184 increased 2-AG concentration in isolated garter snake tissues (via blocking MGL) using a two-way repeated measures ANOVA with time and treatment as within-subjects factors; separate analyses were performed for each incubation temperature. Because all aliquots analyzed for 2-AG were derived from the same pooled sample, we included treatment as a within-subjects factor in these analyses. To determine whether manipulating endocannabinoid signaling over 14 days significantly altered mean courtship score and/or mean locomotion score over time, we used two-way repeated measures ANOVA, with day as the within-subjects factor and treatment as the between-subjects factor. Similarly, to determine whether manipulating endocannabinoids altered the latency to Y-maze choice over time, we used a mixed-model approach with day as the within-subjects factor and treatment as the between-subjects factor.

Latency to Y-maze choice represents an unbounded response variable that was right-censored by a fixed experimental cut-off, with latencies exceeding 20 min recorded as ‘>20 min’. While right-censored data can be analyzed using likelihood-based parametric models, such methods require specification of the underlying distribution of the response variable (Diggle et al., 2002). Given the lack of a well-justified distributional form for these data, latencies were rank transformed prior to analysis using a two-way repeated-measures ANOVA. This rank-based mixed-model ANOVA, established by Conover and Iman (1981) and extended to mixed-model designs by Akritas and Brunner (1997), relaxes distributional assumptions on the original response scale while retaining the factorial design. This approach has been applied in comparable experimental contexts (e.g. Lutterschmidt and Mason, 2008, 2010) and is conceptually aligned with the broader non-parametric framework for longitudinal factorial analysis described by Brunner and Puri (2001), Brunner et al. (2002) and Konietschke et al. (2010), as well as more recent developments summarized by Rubarth et al. (2022).

To determine whether manipulating endocannabinoids significantly altered percentage change in body mass of snakes, we also used a two-way repeated-measures ANOVA with day as the within-subjects factor and treatment as the between-subjects factor. Percentage change in body mass included negative values (mass loss), positive values (mass gain) and true zeros (no change), resulting in a response distribution that did not map cleanly onto distributional families typically used in generalized linear modeling. As such, data were rank transformed prior to analysis with a two-way repeated-measures ANOVA. This approach facilitates time- and treatment-specific comparisons under minimal distributional assumptions.

Finally, to determine whether manipulating endocannabinoids significantly altered the proportion of males choosing worm versus female trails over time, we compared Y-maze choice among treatment groups within each testing day using chi-squared tests. Because Y-maze choice reflects a discrete physiological and/or behavioral transition state rather than a continuum, treatment differences were expected to emerge at specific time points rather than persist across days. Thus, we emphasized identifying day-specific treatment divergence in our analyses rather than estimating a population-averaged treatment effect across time, consistent with Maxwell et al.’s (2018) recommendations for planned comparisons in factorial designs when there is *a priori* knowledge that effects are localized. For days with significant chi-squared results, pairwise differences among treatment groups were further examined using *z*-tests of proportions. Because too few snakes made a choice in the CP-55940 low and high treatment groups, these groups were excluded from statistical analyses of Y-maze choice.

## RESULTS

The endocannabinoids 2-AG and anandamide were identified in brain lipid extracts using LC-MS/MS (Fig. 1A). Qualifier ions of both 2-AG and anandamide indicate the observed concentrations are not the result of matrix effects. Finally, the retention times and qualifier ratios of brain 2-AG and anandamide (Fig. 1B,D) matched the retention times and qualifier ratios of their respective standards (Fig. 1C,E). Across both experiments, the brain concentrations of 2-AG (16.46±0.61 nmol g^−1^) and anandamide (54.92±2.36 pmol g^−1^) in male red-sided garter snakes were approximately 1.5- to 3.5-fold higher than 2-AG (∼4–10 nmol g^−1^) and anandamide (∼15–30 pmol g^−1^) levels found in laboratory rodents (Maccarrone et al., 2002; Patel et al., 2005; Stella et al., 1997).

**Fig. 2.**
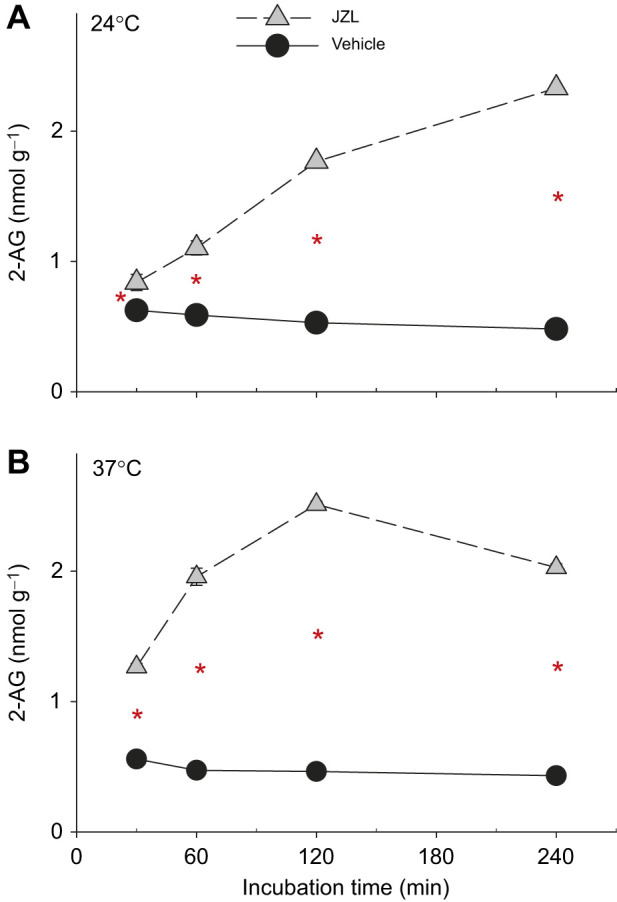
**JZL184 increases 2-AG levels in brain tissue of red-sided garter snake, *in vitro*.** (A,B) Changes in 2-AG measured over time in replicates (*n*=3 at each point) of pooled brain sample treated with JZL184 (a monoacylglycerol lipase inhibitor; 5 μmol l^−1^) or vehicle control and incubated at 24°C or 37°C, respectively. Asterisks indicate significant differences between treatment groups within each time point (*P≤0.004, from a two-way repeated measures ANOVA and Student–Newman–Keuls pairwise multiple comparisons test). Points represent means±1 s.e.m. (error bars hidden by symbols).

Prior to experiment 3, we evaluated whether JZL184 increases 2-AG concentration in garter snake tissue, as it is known to do in mice (Pan et al., 2009) and humans (Long et al., 2009; but note that this study showed JZL184's effect is not consistent across all mammals). At 24°C, the concentration of 2-AG in replicates of pooled brain sample treated with JZL184 (5 μmol l^−1^) was significantly higher than that in brains treated with a vehicle control (Fig. 2A; *F*_1,6_=998.803, *P*=0.001, η^2^=0.568). Additionally, 2-AG concentrations were significantly different across incubation time (*F*_3,6_=222.878, *P*<0.001, η^2^=0.174) and a significant interaction was observed between treatment and incubation time (*F*_3,6_=788.006, *P*<0.001, η^2^=0.253; from a two-way repeated measures ANOVA). Similarly, at 37°C, the concentration of 2-AG in replicates of pooled brain sample treated with JZL184 (5 μmol l^−1^) was significantly higher than that in brains treated with a vehicle control (Fig. 2B; *F*_1,6_=5188.980, *P*<0.001, η^2^=0.840) and 2-AG concentrations were significantly different across incubation times (*F*_3,6_=157.552, *P*<0.001, η^2^=0.066); a significant interaction was observed between treatment and incubation time (*F*_3,6_=157.841, *P*<0.001, η^2^=0.092; from a two-way repeated measures ANOVA). Taken together, these experiments indicate that JZL184 inhibits MGL in the red-sided garter snake to increase endogenous levels of 2-AG.

### Experiment 1: variation in endocannabinoids related to behavioral phenotype

**Fig. 3.**
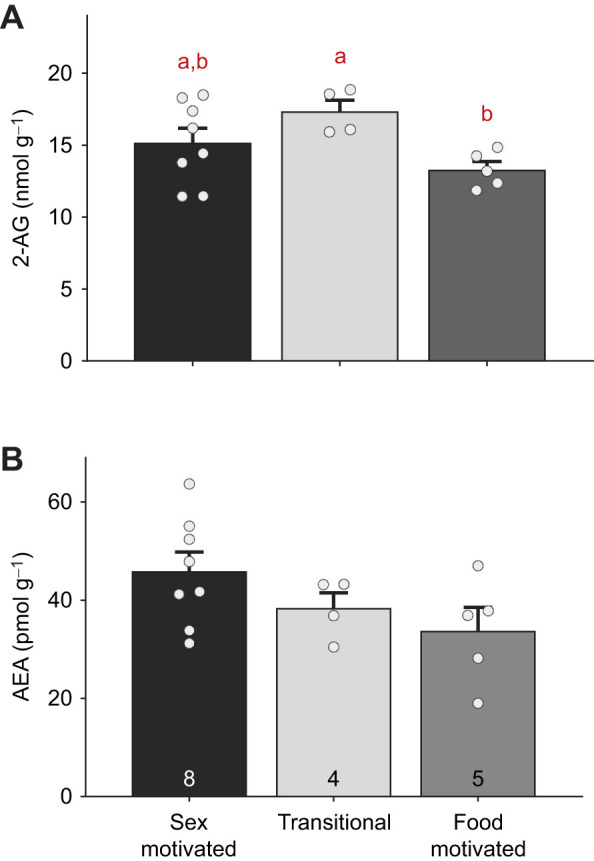
**Brain 2-AG, but not anandamide, varies with male behavioral phenotype as red-sided garter snakes transition from sex to feeding behavior.** (A,B) Whole-brain levels of 2-AG and anandamide (AEA), respectively. Lowercase letters indicate significant differences among phenotypes (*P*=0.044; from a one-way ANOVA with a Student–Newman–Keuls multiple comparisons test). Bars represent means+1 s.e.m.; circles represent individual data points. Numbers along the *x*-axis indicate *n* values for each group.

Significant variation in whole-brain 2-AG concentration was observed across behavioral phenotypes (Fig. 3A; *F*=3.617, d.f.=2, *P*=0.054, η^2^=0.341; from a one-way ANOVA). In contrast, whole-brain anandamide concentration did not vary with behavioral phenotype (Fig. 3B; *F*=2.352, d.f.=2, *P*=0.132, η^2^=0.252; from a one-way ANOVA).

### Experiment 2: variation in endocannabinoids related to sex and migratory behavior

**Fig. 4.**
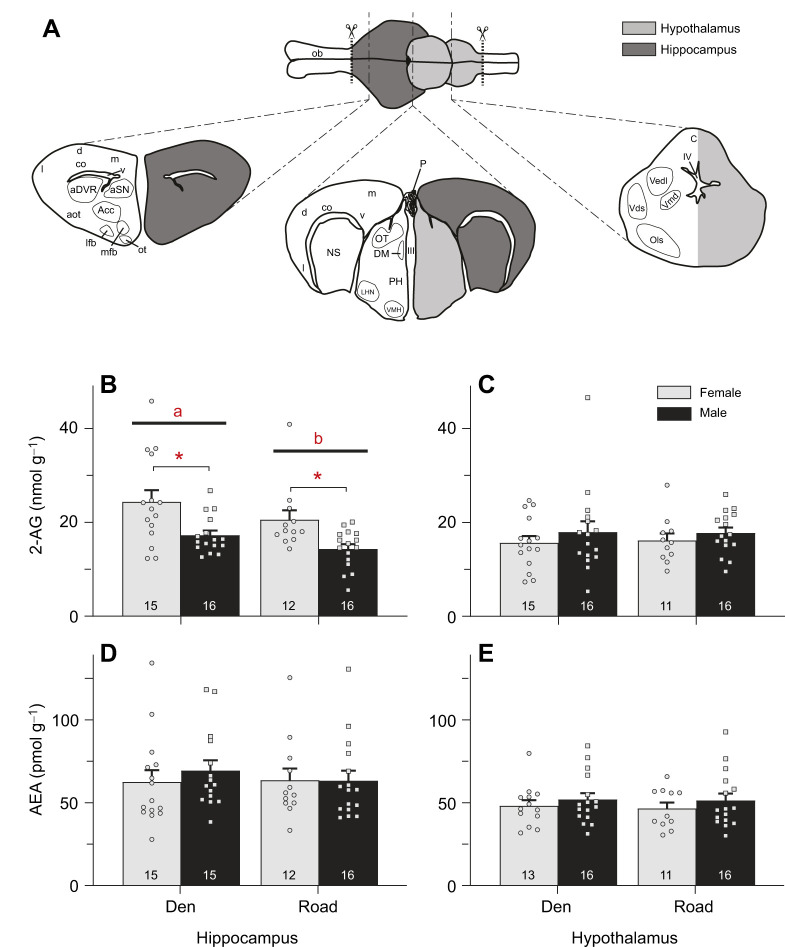
**Migratory red-sided garter snakes have lower 2-AG concentrations in the hippocampus compared with non-migratory snakes.** (A) The diencephalon, where the primary neuroendocrine structure is the hypothalmus, and the telencephalon, which includes the functional homolog of the hippocampus and medial amygdala, were microdissected from male and female garter snakes. We refer to these structures as the hypothalamus (light gray shading) and hippocampus (dark gray shading), respectively. (B,C) 2-AG in the hippocampus and hypothalamus, respectively. (D,E) Anandamide (AEA) in the hippocampus and hypothalamus, respectively. Female (light gray bars, circles) and male (dark gray bars, squares) snakes were collected from a den prior to migration and from a road during the initial stages of migration. Lowercase letters indicate significant differences between snakes collected from the den versus road (*P*=0.038, main effects from a two-way ANOVA). Asterisks above brackets indicate significant differences between sexes within each collection site (*P*≤0.011, from a Student–Newman–Keuls pairwise multiple comparisons test). Bars are means+1 s.e.m. Numbers along the *x*-axis indicate *n* values for each group. Brain region abbreviations: Acc, nucleus accumbens; aDVR, anterior dorsal ventricular ridge; aot, accessory olfactory tract; aSN, anterior septal nucleus; C, cerebellum; co, cortex; d, dorsal cortex; DM, dorsomedial thalamic nucleus; III, third ventricle; IV, fourth ventricle; l, lateral cortex; lfb, lateral forebrain bundle; LHN, lateral posterior hypothalamic nucleus; m, medial cortex; mfb, medial forebrain bundle; NS, nucleus sphericus; ob, olfactory bulb; Ols, superior olive; ot, olfactory tubercle; OT, optic tectum; P, pineal gland; PH, periventricular hypothalamic nucleus; Vds, descending nucleus of the trigeminal nerve; v, lateral ventricle; Vedl, dorsolateral vestibular nucleus; Vmd, dorsal motor nucleus of the trigmeminal nerve; VMH, ventromedial hypothalamic nucleus. (Brain schematics adapted from Maine et al., 2014.)

The concentration of 2-AG in the hippocampus varied significantly with sex (Fig. 4A,B; *F*_1,55_=16.309, *P*<0.001, η^2^=0.212) and migratory status (*F*_1,55_=4.496, *P*=0.038, η^2^=0.058); the interaction between factors was not statistically significant (*F*_1,55_=0.192, *P*=0.663, η^2^=0.002; from a two-way ANOVA). In the hypothalamus, no such differences in 2-AG were observed with sex (Fig. 4C; *F*_1,54_=1.040, *P*=0.312, η^2^=0.019) or migratory status (*F*_1,54_=0.371, *P*=0.545, η^2^=0.007); a significant interaction was not observed between sex and migratory status (*F*_1,54_<0.001, *P*=0.990, η^2^=0.000; from a two-way ANOVA).

The concentration of anandamide in the hippocampus did not vary significantly with sex (Fig. 4A,D; *F*_1,54_=0.676, *P*=0.415, η^2^=0.012) or migratory status (*F*_1,54_=0.100, *P*=0.753, η^2^<0.002); the interaction between factors was not statistically significant (*F*_1,54_=0.734, *P*=0.395, η^2^=0.013; from a two-way ANOVA). In the hypothalamus, anandamide concentration did not vary significantly with sex (Fig. 4E; *F*_1,52_=2.050, *P*=0.158, η^2^=0.038) or migratory status (*F*_1,52_=0.009, *P*=0.926, η^2^=0.000); a significant interaction was not observed between sex and migratory status (*F*_1,52_=0.243, *P*=0.624, η^2^=0.004; from a two-way ANOVA).

### Experiment 3: effects of endocannabinoid signaling on courtship and feeding behavior

Our experiment included a low and high dose of all treatments to assess dose effects. However, 33% (*n*=6) of snakes showed an adverse response to the high dose of AM251 (150 μg; approximately 5 mg kg^−1^) after 6 daily injections, including reduced muscle tone and a slow righting response. We therefore immediately isolated all snakes in this treatment group, discontinued the treatment and eliminated the treatment group from this experiment. Snakes did not display adverse responses to the low dose of AM251 (15 μg; approximately 0.5 mg kg^−1^). Arguably, the severe response snakes had to the high dose of AM251 suggests endocannabinoid signaling via CB1 receptor plays a critical role in their ability to survive.

**Fig. 5.**
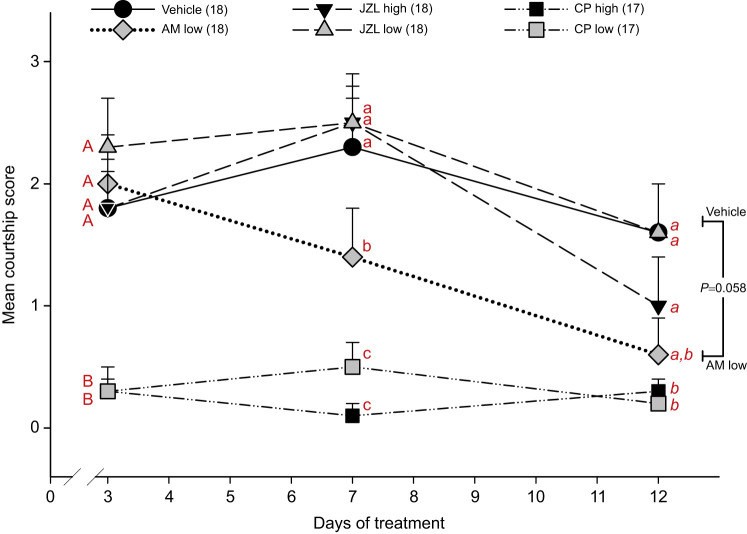
**Blocking endocannabinoid signaling with AM251 decreases courtship behavior of red-sided garter snakes.** Changes in mean courtship scores from a 1 h courtship trial among treatment groups: vehicle; cannabinoid 1 (CB1) receptor antagonist AM251 (AM low; ∼0.5 mg kg^−1^); 2-AG's degradation enzyme inhibitor JZL184 (JZL low or high; ∼1.6 or 16 mg kg^−1^); cannabinoid receptor agonist CP-55940 (CP low or high; ∼0.1 or 1 mg kg^−1^). Significant differences are indicated among treatment groups at 3 days (uppercase letters indicate *P*≤0.002), 7 days (lowercase letters indicate *P*≤0.054) and 12 days (lowercase italicized letters indicate *P*≤0.012) of treatment; statistics are from a two-way repeated measures ANOVA with a Student–Newman–Keuls pairwise multiple comparisons test. The bracket indicates a near-significant difference between vehicle and AM low at day 12. Points represent means+1 s.e.m. Numbers in parentheses within the figure key indicate *n* values for each group.

**Fig. 6.**
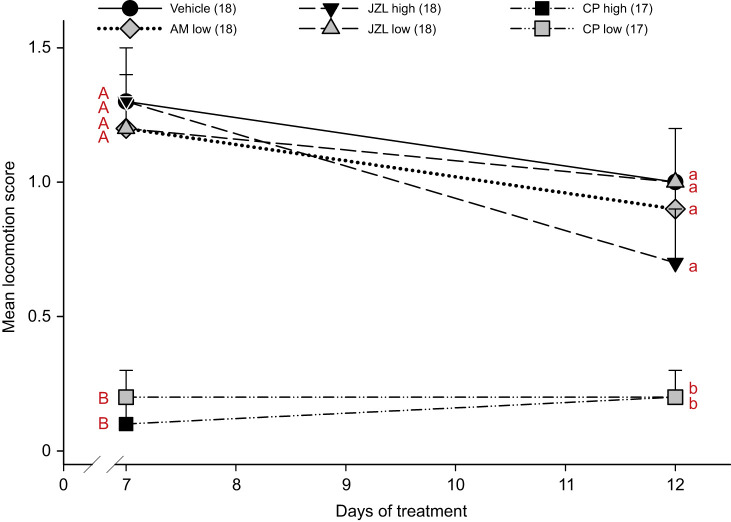
**Activation of cannabinoid receptors by CP-55940 decreases locomotion of red-sided garter snakes, but increasing endogenous 2-AG with JZL184 does not.** Change in mean locomotion scores among treatment groups: vehicle; CB1 receptor antagonist AM251 (AM low; ∼0.5 mg kg^−1^); 2-AG's degradation enzyme inhibitor JZL184 (JZL low or high; ∼1.6 or 16 mg kg^−1^); cannabinoid receptor agonist CP-55940 (CP low or high; ∼0.1 or 1 mg kg^−1^). Significant differences are indicated among treatment groups at 7 days (uppercase letters indicate *P*<0.001) and 12 days (lowercase letters indicate *P*≤0.011) of treatment; statistics are from a two-way repeated measures ANOVA with a Student–Newman–Keuls pairwise multiple comparisons test. Points represent means+1 s.e.m. Numbers in parentheses in the figure key indicate *n* values for each group.

Mean courtship scores showed a statistically significant interaction between treatment group and days of treatment (Fig. 5; *F*_10,200_=1.86, *P*=0.053, η^2^=0.031; from a two-way repeated measures ANOVA); scores varied significantly among the treatment groups (*F*_5,200_=13.833, *P*<0.001, η^2^=0.246) and with days of treatment (*F*_2,200_=11.612, *P*<0.001, η^2^=0.038). Mean locomotion score also varied significantly among drug treatment groups (Fig. 6; *F*_5,100_=20.689, *P*<0.001, η^2^=0.381) and with days of treatment (*F*_1,100_=13.700, *P*<0.001, η^2^=0.028); no significant interaction was observed between factors (*F*_5,100_=2.117, *P*=0.069, η^2^=0.021; from a two-way repeated measures ANOVA).

**Fig. 7.**
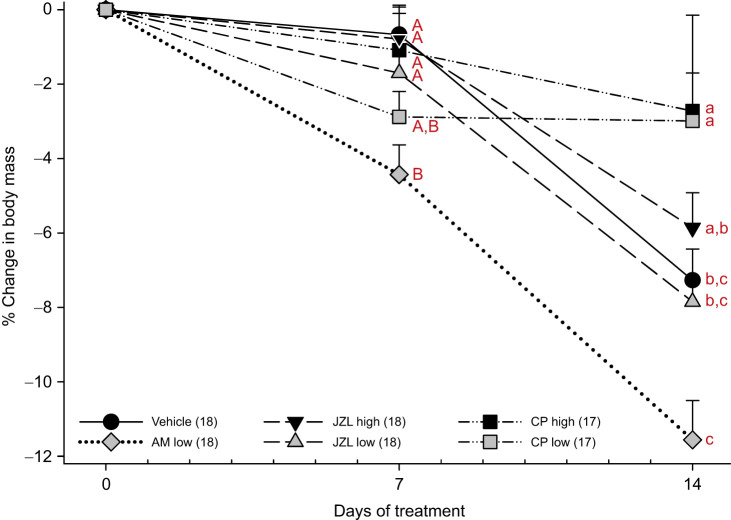
**Blocking endocannabinoid signaling with AM251 increases loss of body mass in aphagic red-sided garter snakes.** Percentage change in body mass among treatment groups: vehicle; CB1 receptor antagonist AM251 (AM low; ∼0.5 mg kg^−1^); 2-AG's degradation enzyme inhibitor JZL184 (JZL low or high; ∼1.6 or 16 mg kg^−1^); cannabinoid receptor agonist CP-55940 (CP low or high; ∼0.1 or 1 mg kg^−1^). Significant differences are indicated among treatment groups at 7 days (uppercase letters indicate *P*≤0.011) and 14 days (lowercase letters indicate *P*≤0.045) of treatment; statistics are from a two-way repeated measures ANOVA with a Student–Newman–Keuls pairwise multiple comparisons test. Points represent means+1 s.e.m. Numbers in parentheses within the figure key indicate *n* values for each group.

Percentage change in body mass showed a statistically significant interaction between treatment group and days of treatment (Fig. 7; *F*_10,200_=4.873, *P*<0.001, η^2^=0.055; from a two-way repeated measures ANOVA). Additionally, percentage change in body mass varied significantly among treatments groups (*F*_5,200_=3.237, *P*=0.009, η^2^=0.042) and with days of treatment (*F*_2,200_=184.064, *P*<0.001, η^2^=0.412).

**Fig. 8.**
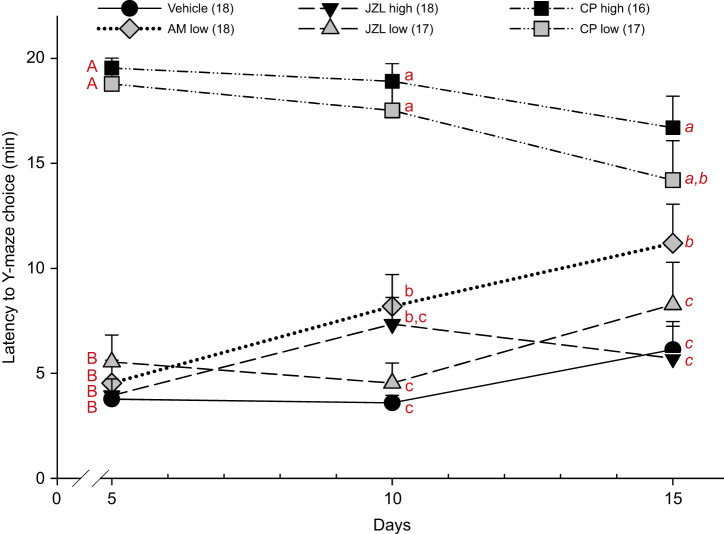
**Manipulating endocannabinoid signaling increases latency to Y-maze choice in red-sided garter snakes.** Changes to mean latency to Y-maze choice among treatment groups: vehicle; CB1 receptor antagonist AM251 (AM low; ∼0.5 mg kg^−1^); 2-AG's degradation enzyme inhibitor JZL184 (JZL low or JZL high; ∼1.6 or 16 mg kg^−1^); cannabinoid receptor agonist CP-55940 (CP low or high; ∼0.1 or 1 mg kg^−1^). Significant differences among treatment groups at 5 days (uppercase letters indicate *P*<0.001), 10 days (lowercase letters indicate *P*≤0.026) and 15 days (lowercase italicized letters indicate *P*≤0.042) of treatment; statistics are, from a two-way repeated measures ANOVA with a Student–Newman–Keuls pairwise multiple comparisons test. Points represent means+1 s.e.m. Numbers in parentheses within the figure key indicate *n* values for each group.

Latency to Y-maze choice showed a statistically significant interaction between treatment group and days of treatment [Fig. 8; *F*_9.017,171.326_=3.065, *P*=0.002, η^2^=0.048; from a two-way repeated measures ANOVA with a Greenhouse–Geisser correction (ϵ=0.902)]. Latency to Y-maze choice varied significantly among treatment groups (*F*_5,171.326_=44.868, *P*<0.001, η^2^=0.456) but not with days of treatment (*F*_1.803,171.326_=2.04, *P*=0.138, η^2^=0.006).

**Fig. 9.**
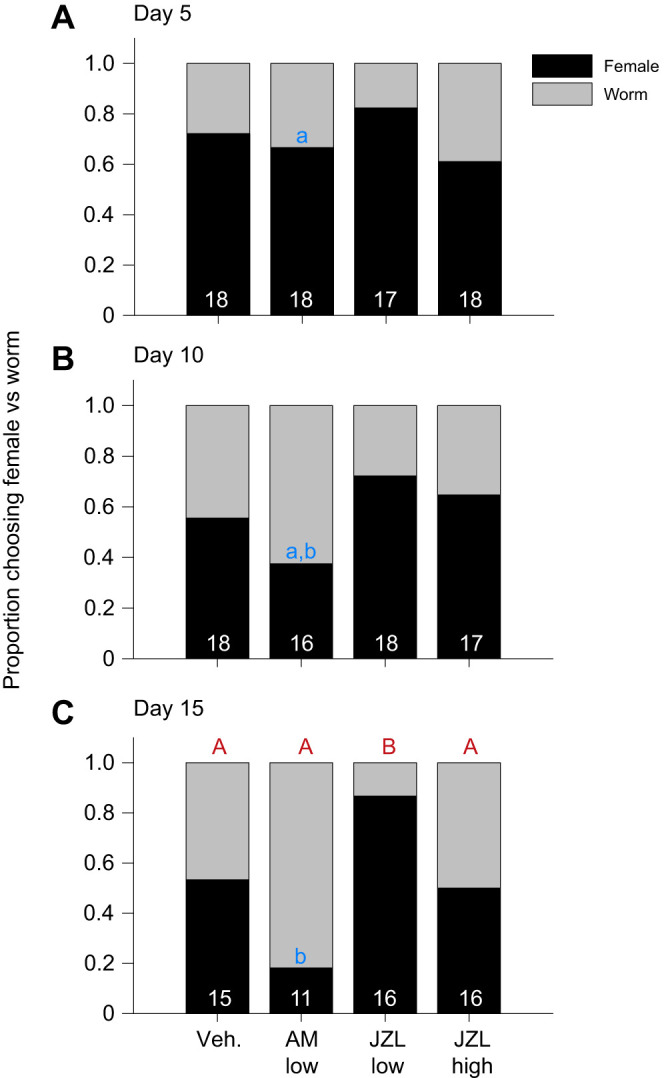
**Increasing endogenous 2-AG with JZL184 extends sex-motivated behavior, while blocking endocannabinoid signaling with AM251 advances the transition to food-motivated behavior in red-sided garter snakes.** (A–C) Changes in the proportion of snakes choosing female (black) or worm (gray) trails on the Y-maze on days 5, 10 and 15 respectively, among treatment groups: vehicle (Veh.); CB1 receptor antagonist AM251 (AM low; ∼0.5 mg kg^−1^); 2-AG's degradation enzyme inhibitor JZL184 (JZL low or JZL high; ∼1.6 or 16 mg kg^−1^). Significant differences among treatment groups within day 15 (uppercase letters indicate *P*≤0.046) and across time within AM251 treatment only (lowercase letters indicate *P*=0.011), from a chi-squared test followed by *z*-tests for comparisons between treatments. Numbers along the *x*-axes indicate *n* values for each group.

The proportion of snakes that chose female versus worm trails among drug treatments did not vary significantly on day 5 (Fig. 9A; χ^2^_3_=2.061, *P*=0.560, *V*=0.170) or on day 10 (Fig. 9B; χ^2^_3_=4.612, *P*=0.203, *V*=0.259). However, significant variation was observed among drug treatments on day 15 (Fig. 9C; χ^2^_3_=12.243, *P*=0.007, *V*=0.463; from a chi-squared test). We further determined that the proportion of snakes choosing the female trail when treated with the low dose of AM251 significantly decreased across days (χ^2^_2_=6.985, *P*=0.030, *V*=0.394; from a chi-squared test); this was not the case for snakes treated with vehicle.

## DISCUSSION

The endocannabinoid system's ability to regulate the neural circuits that control feeding (e.g. Koch, 2017), sex behavior (e.g. Rodríguez-Manzo and Canseco-Alba, 2023) and synaptic plasticity (e.g. Winters and Vaughan, 2021) makes it a strong candidate for mediating seasonal transitions in behavior. Our results show that brain levels of 2-AG, but not anandamide, change significantly during the seasonal transition from reproduction to migration and feeding behavior. Moreover, manipulating endocannabinoid levels altered the timing of this behavioral transition in male snakes. Together, our results support a role for endocannabinoids in mediating the seasonal transition from reproduction to self-maintenance behavior.

### Variation in endocannabinoids in the brain

Many studies have established the orexigenic effects of endocannabinoids within the brain in laboratory rodents. In this study, we found that male snakes exhibiting a food-motivated phenotype had significantly lower whole brain 2-AG compared with snakes that were transitioning from sex-motivated to food-motivated behavior (Fig. 3). Although our data do not align with the orexigenic effects of endocannabinoids observed in many animals, there is precedence for variation across taxa. For example, exogenous endocannabinoid treatment in fruit flies significantly inhibited food intake, suggesting endocannabinoids sometimes have anorectic effects (He et al., 2021). An alternative explanation is provided by a closer assessment of the behavioral phenotypes exhibited by snakes during this seasonal life-history transition. Specifically, both transitional snakes and food-motivated snakes showed the same behavioral phenotype on the Y-maze (i.e. they chose the trail representing a feeding opportunity). In contrast, transitional snakes continued to court females in mating trials, while food-motivated snakes did not. We speculate that the significantly lower 2-AG levels in food-motivated snakes are more strongly associated with the seasonal decline in reproductive behavior rather than the activation of feeding behavior. Our data from pharmacologically increasing 2-AG levels of male snakes with JZL184 (Fig. 9), which prolonged appetitive reproductive behavior, support this hypothesis. However, it must be noted that whole-brain analyses cannot capture variation across different brain regions that may contribute differentially to seasonal changes in behavior.

We have begun to address this question by examining endocannabinoid levels in different regions of the brain. Similar to the data from our whole-brain analyses, we found that male and female snakes collected from the den where spring mating occurs had significantly higher 2-AG levels in the hippocampus compared with snakes collected early in their migration to summer feeding grounds (Fig. 4). There were no changes in 2-AG levels within the hypothalamus. Thus, the decrease in whole-brain 2-AG in food-motivated snakes compared with transitional snakes was likely driven by changes in the hippocampus. While the hypothalamus is the primary control region of both sex and feeding behavior, the hippocampus plays a modulatory role, particularly on the hypothalamus–pituitary–adrenal (HPA) axis, where it exerts negative feedback on glucocorticoid signaling via neurons that signal into the paraventricular nucleus (reviewed by Ulrich-Lai and Herman, 2009). These results are significant because prior studies in this population of garter snakes found a similar pattern in plasma glucocorticoids, with significantly higher baseline glucocorticoids observed in snakes courting at the breeding grounds compared with those migrating toward summer feeding grounds (Cease et al., 2007).

While the interaction between the HPA axis and endocannabinoid system is not fully understood, evidence strongly supports a bi-directional relationship. That is, not only does glucocorticoid signaling mobilize endocannabinoids to exert many of its effects (Balsevich et al., 2017), but increased endocannabinoids, specifically 2-AG, exert negative feedback inhibition on the HPA axis to terminate an acute stress response (Morena et al., 2016). Moreover, a relationship between hippocampal endocannabinoids and glucocorticoid signaling has been established. In male rats, infusion of the CB1 receptor antagonist AM251 into the hippocampus blocked the freezing effects of glucocorticoid treatment (Atsak et al., 2012). Similarly, infusion of the CB1 receptor agonist WIN55,212-2 produced the same effects on freezing behavior as glucocorticoid treatment (Atsak et al., 2012). Finally, both sustained glucocorticoid elevation and repeated stress exposure increased 2-AG in the hippocampus of male mice (Bowles et al., 2012; Dubreucq et al., 2012). Thus, the differences in hippocampal 2-AG reported here are likely involved in mediating the known seasonal changes in both the HPA axis and responses to glucocorticoids as male and female snakes transition from reproduction to feeding (Cease et al., 2007; Dayger and Lutterschmidt, 2016, 2017; Lutterschmidt and Maine, 2014; Moore et al., 2001). While endocannabinoid receptors are known to be expressed throughout the vertebrate diencephalon and telencephalon (Cottone et al., 2005; Hollis et al., 2006; Tsou et al., 1998), the exact distribution in reptiles has not yet been determined. Additional region-specific studies are needed to further elucidate whether the observed differences in 2-AG are localized to the reptilian homolog of the hippocampus, amygdala and/or other telencephalon regions.

Although glucocorticoids and endocannabinoids are both implicated in metabolic regulation, including insulin signaling and glucose metabolism, their functional relationship remains complex and context dependent (reviewed by Kuo et al., 2015; see also Romero and Beattie, 2022). Despite migrating red-sided garter snakes having lower baseline glucocorticoid (Cease et al., 2007) and hippocampal 2-AG (this study) concentrations than snakes at the breeding grounds, they showed no significant differences in liver glycogen or adipocyte area (Wilson and Lutterschmidt, 2020). It is likely that the seasonal transition from reproduction to feeding behavior in this species is associated with other metabolic factors that have not yet been examined. For example, in western terrestrial garter snakes (*Thamnophis elegans*), variation in insulin-like growth factors (IGF-1 and -2) is associated with life-history ecotypes and trade-offs between reproduction and self-maintenance, including litter size, growth rate and life-span (Reding et al., 2016; Schwartz and Bronikowski, 2016; Sparkman et al., 2009). Furthermore, crosstalk between endocannabinoid and insulin/IGF signaling pathways has been reported in mammals. Specifically, the ability of IGF-1 to increase the rate of both spontaneous and excitatory postsynaptic currents depends on endocannabinoid signaling, as blocking CB1 receptors abolishes the effects of IGF-1 (Bálint et al., 2021). Further research exploring the role of insulin/IGF signaling pathways during spring migration would help in understanding how glucocorticoids, endocannabinoids and metabolic factors interact to regulate the transition from mating to feeding behavior.

The energetic profiles associated with life-history transitions may very well emerge downstream of, rather than precede, changes in physiology and behavior. This temporal distinction could be particularly relevant in a dissociated breeder such as the red-sided garter snake, in which peak circulating sex steroid hormones and gamete maturation do not occur during the mating season (reviewed by Crews, 1984; Crews et al., 1984). Although plasma concentrations of sex steroid hormones are low and/or decreasing during the mating season (reviewed by Lutterschmidt, 2012), this dissociated reproductive pattern does not preclude trans-seasonal interactions between endocannabinoids and the hypothalamus–pituitary–gonad (HPG) axis from regulating seasonal changes in reproductive behavior. While data are limited in non-mammalian vertebrates, evidence from mammals suggests endocannabinoid signaling can reduce the release of hypothalamic gonadotropin-releasing hormone (GnRH), resulting in decreased circulating testosterone (reviewed by Du Plessis et al., 2015) and/or estradiol (reviewed by Santoro et al., 2021). Our results, in part, align with these effects, as we found that 2-AG concentrations in the hippocampus of male and female snakes are elevated during the mating season, when testosterone concentrations are low and estradiol is undetectable (reviewed by Lutterschmidt, 2012; Dayger et al., 2013). Moreover, female snakes had significantly higher 2-AG in the hippocampus compared with male snakes at both the den and road. Although little is known about how the actions of sex steroids impact or interact with hippocampal endocannabinoid levels, one electrophysiology study offers clues. In female guinea pigs, estradiol-induced suppression of inhibitory postsynaptic currents in hippocampal glutamatergic neurons was blocked by pretreatment with AM251 and occluded with pretreatment of a cannabinoid receptor agonist (Huang and Woolley, 2012). Additionally, Huang and Woolley (2012) found that increasing anandamide levels by inhibiting its degradation enzyme mirrored the effects of the cannabinoid receptor agonist. Future studies should directly investigate how changes in endocannabinoids within specific brain regions relate to seasonal modulation of the HPG axis and patterns of sex steroid hormones. Comparing these relationships in both associated and dissociated breeders would be advantageous in understanding the role that sex steroid hormones play in endocannabinoid–HPG axis interactions.

Unlike 2-AG, no differences in anandamide were observed with behavioral phenotype, migratory status or sex. In rats, anandamide is 170-fold less concentrated than 2-AG in the brain (Stella et al., 1997). In line with the literature, brain anandamide concentrations in garter snakes were approximately 380-fold lower than 2-AG levels. Sustained exposure to either elevated glucocorticoids or repeated stress is known to decrease anandamide concentrations in the prefrontal cortex, amygdala, hippocampus and hypothalamus (Gray et al., 2016; Hill et al., 2010). Given that garter snakes have elevated baseline glucocorticoids during winter dormancy (Lutterschmidt et al., 2022) and spring breeding (Cease et al., 2007; Krohmer et al., 1987; Moore et al., 2001; Moore and Mason, 2001; Whittier et al., 1987), it is possible that the substantial difference between 2-AG and anandamide levels we observed is seasonally specific. Comparing 2-AG and anandamide levels during other times of year could provide further support for the relationship between glucocorticoids and endocannabinoids.

### Behavioral responses to pharmacological manipulation of endocannabinoids

We directly tested the hypothesis that endocannabinoids mediate the seasonal life-history transition from reproduction to feeding by examining whether pharmacological manipulation of endocannabinoid signaling altered the expression or timing of sex and/or feeding behavior. Behavioral data from male snakes treated daily for 14 days with vehicle, AM251 (CB1 receptor antagonist), JZL184 (inhibitor of MGL, the 2-AG degradation enzyme) or CP-55940 (cannabinoid receptor agonist) revealed two key findings: (1) CB1 receptors are necessary for courtship behavior, as blocking them with AM251 reduced courtship behavior and resulted in an early transition to feeding behavior; and (2) increasing 2-AG levels with JZL184 was sufficient to extend the duration of appetitive reproductive behavior and delay the transition to feeding behavior. Interestingly, agonizing cannabinoid receptors globally produced a different effect compared with increasing endogenous 2-AG levels with JZL184. Our results suggest that the influence of endocannabinoids on sex and feeding behavior is mediated by brain regions that are already producing 2-AG, with 2-AG likely acting through CB1 receptor signaling.

Importantly, although AM251 treatment significantly reduced courtship behavior (Fig. 5), it did not alter locomotor activity (Fig. 6), indicating the effects on courtship and the early shift to food-motivated behavior (Fig. 9) were not the result of impaired movement. Similar to our findings, inhibiting glucocorticoid synthesis with metyrapone also caused an early transition to food-motivated behavior but did not inhibit courtship behavior (Lutterschmidt and Maine, 2014). It is possible that the observed influence of endocannabinoids on the seasonal transition from sex to feeding behavior results from their effects on other neuroendocrine factors, including, but not limited to, glucocorticoids. Notably, while Coddington et al. (2007) found that treatment with a similar CB1 receptor antagonist, AM281, had no effect on the proportion of rough-skinned newts displaying clasping courtship behavior, it significantly attenuated the inhibitory effects of glucocorticoids on courtship.

While increasing 2-AG with the low dose of JZL184 extended the period during which snakes pursued reproductive opportunities, it had no effect at either dose on the intensity of courtship behavior (Fig. 5) or locomotor activity (Fig. 6). Although the design of our courtship trials reduces the possibility of ceiling effects, it is possible that treatment with JZL184 could not increase courtship intensity beyond the levels observed in control snakes during the spring mating season. Nevertheless, our data support a role for 2-AG in facilitating reproductive behavior.

Although there is precedence for neuromodulators gating sexual and social behavior, no studies other than the present one and Coddington et al. (2007) have investigated the association between endocannabinoids and sex behavior in any wild animal. However, a limited number of studies on laboratory rodents have been done (reviewed by Rodríguez-Manzo and Canseco-Alba, 2023). Less is known about repeated administration, but acute administration of exogenous 2-AG appears to have no effect on the sexual behavior of non-satiated male rats (Canseco-Alba and Rodriguez-Manzo, 2019). These results mirror our findings that JZL184 did not alter male courtship scores. However, Canseco-Alba and Rodriguez-Manzo (2019) further showed that 2-AG treatment significantly increased the percentage of sexually satiated rats (tested 24 h after sexual exhaustion) that display mounting behavior, intromissions, ejaculations and resumption of copulation after ejaculation, effectively extending their reproductive behavior beyond their refractory period. Although we did not observe sexual exhaustion in our study, the results of Canseco-Alba and Rodriguez-Manzo (2019) are comparable to our findings that JZL184 extends the duration of appetitive sex behavior compared with vehicle-treated snakes. Puzzlingly, rat studies also show that treatment with a single dose of AM251 (1, 3 or 5 mg kg^−1^) facilitates sex behavior, as treated rats display reduced ejaculation latency and an increased number of ejaculations (Canseco-Alba and Rodríguez-Manzo, 2014; Gorzalka et al., 2008). More studies are needed, in both wild and non-wild animals, to fully parse out the role of endocannabinoids in reproductive behavior.

Unexpectedly, snakes treated with low dose AM251 lost significantly more body mass than snakes treated with vehicle for 7 and 14 days (Fig. 7). These results are supported by some previous studies, where AM251 has been shown to reduce body mass in a rodent model of obesity, in part due to decreased food intake and increased energy expenditure (Merroun et al., 2013; O'Keefe et al., 2022). However, the red-sided garter snakes used in this study are in a prolonged fasted state during winter dormancy and spring mating, and they showed neither a decrease in food intake nor an increase in energy expenditure (as assessed by locomotor activity) in response to AM251 treatment. Thus, further studies are needed to understand the mechanism by which CB1 receptor antagonism influences metabolism and body mass.

In contrast to JZL184, treatment with the CB1 receptor agonist CP-55940 significantly inhibited locomotion (Fig. 6), making it impossible to determine whether the observed decrease in courtship scores (Fig. 5) are the result of a change in locomotor activity, appetitive sex behavior or both. Importantly, this depression of locomotor activity mimics the effects of CP-55940 in mice and rats (Costa et al., 1996; Hodges et al., 2020) as well as the effects of the cannabis extract THC on rats (Whitlow et al., 2002), supporting the presence of an evolutionarily conserved endocannabinoid system across vertebrates. CP-55940 also significantly increased snakes' latency to make a choice on the Y-maze (Fig. 9) and significantly decreased the rate of body mass loss compared with vehicle-treated snakes after 14 days of treatment (Fig. 7), both the likely result of reduced locomotor activity. We hypothesize that these effects differed from those of JZL184 due to differences in the dose–response curves (i.e. the lowest dose of CP-55940 agonized CB1 receptors more than the lowest dose of JZL184) and/or their mechanisms of action (i.e. JZL184 increases 2-AG levels where 2-AG already exists in the body, while CP-55940 acts on both CB1 and CB2 receptors globally). Future experiments using CP-55940 at lower doses would aid in addressing this hypothesis. Our results highlight the significance of region-specific activity of CB1 receptors and suggest the effects of 2-AG on the transition from sex to feeding behavior depend on the brain region being influenced.

As the molecular mechanisms of 2-AG signaling are highly conserved across chordates and many invertebrates (Elphick, 2012), these results reveal endocannabinoid functions that are fundamentally relevant to all organisms, particularly those with seasonal behaviors. While species-level variation is likely, we expect that differences in endocannabinoid function might mirror the variation observed in the regulation of glucocorticoids across species, where the anatomy and physiological properties of the HPA axis are conserved but their effects vary across seasons and/or species (Romero and Gormally, 2019). Exploring how endocannabinoids change across different body tissues over time, particularly across seasons, might reveal more about their role in life-history transitions. Ultimately, our data suggest there is a larger evolutionary role to be discovered for endocannabinoids in mediating behavioral output.

## Supplementary Material

10.1242/jexbio.251845_sup1 Supplementary information
