# Supplementary material for: The endocannabinoid 2-arachidonoylglycerol mediates seasonal life-history transitions in a wild reptile
Source: J Exp Biol. 2026 Jul 6;229(13):jeb251845. doi: 10.1242/jeb.251845 (PMC13380979; doi:10.1242/jeb.251845)
Supplement: Supplementary information [file jexbio-229-251845-s1.pdf]

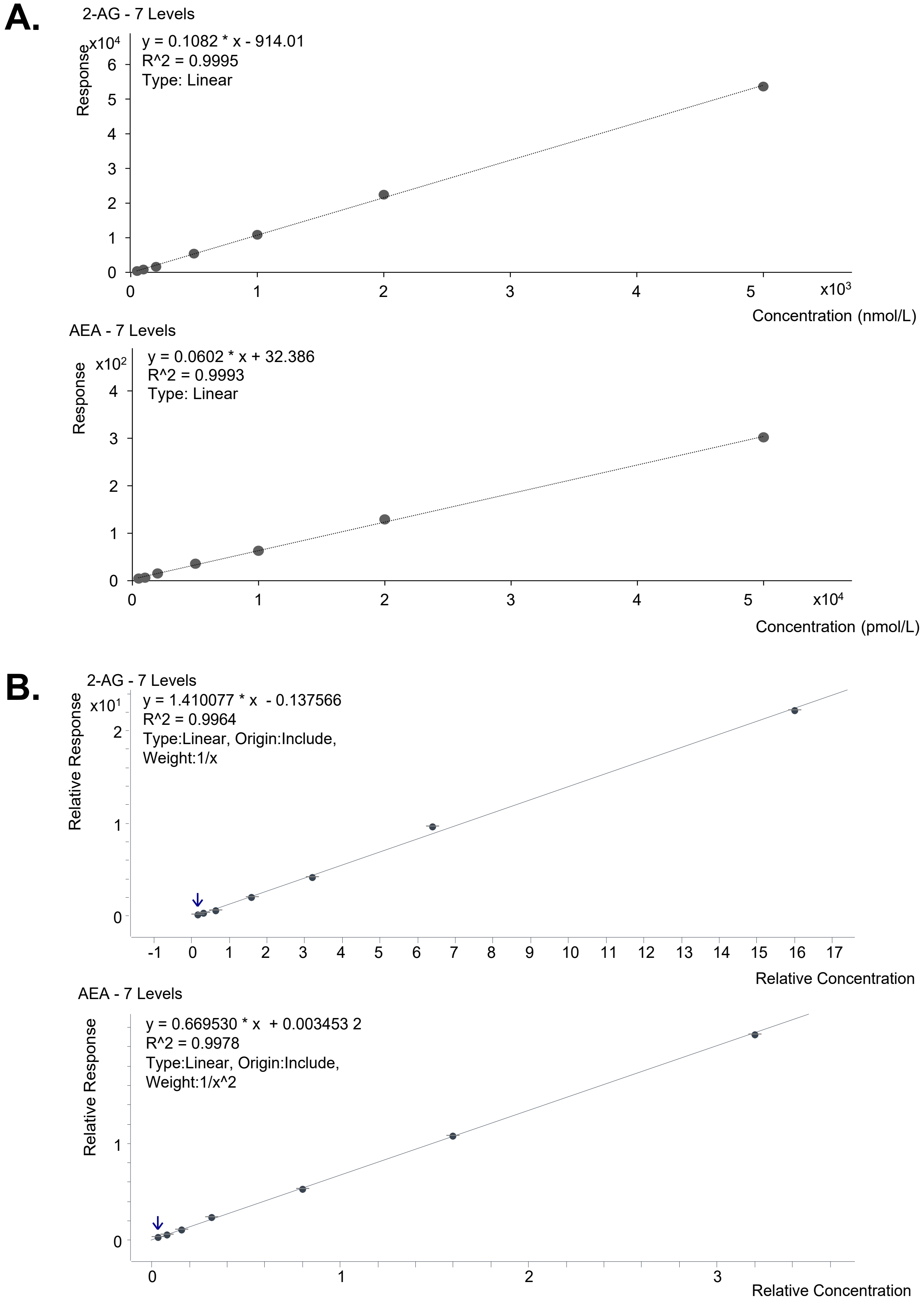
A.
2-AG - 7 Levels
y = 0.1082 * x - 914.01
R^2 = 0.9995
Type: Linear
Response
x10^4
Concentration (nmol/L)
x10^3
AEA - 7 Levels
y = 0.0602 * x + 32.386
R^2 = 0.9993
Type: Linear
Response
x10^2
Concentration (pmol/L)
x10^4
B.
2-AG - 7 Levels
y = 1.410077 * x - 0.137566
R^2 = 0.9964
Type:Linear, Origin:Include,
Weight:1/x
Relative Response
x10^1
Relative Concentration
AEA - 7 Levels
y = 0.669530 * x + 0.003453 2
R^2 = 0.9978
Type:Linear, Origin:Include,
Weight:1/x^2
Relative Response
Relative Concentration

Fig. S1. Calibration curves for 2-arachidonoylglycerol (2-AG) and anandamide (AEA) without their [^{2}H]-containing internal standards (A), as used in Experiment 1 and with their [^{2}H]-containing internal standards (B), as used in Experiment 2.

Journal of Experimental Biology • Supplementary information
